# Effects of aggregate sizes on the performance of laterized concrete

**DOI:** 10.1038/s41598-023-50998-1

**Published:** 2024-01-03

**Authors:** Joseph O. Ukpata, Desmond E. Ewa, Nwajei Godwin Success, George Uwadiegwu Alaneme, Obeten Nicholas Otu, Bamidele Charles Olaiya

**Affiliations:** 1 Department of Civil Engineering, University of Cross River State, Calabar, Nigeria; 2 Department of Civil Engineering, Kampala International University, Kampala, Uganda; 3 Department of Civil Engineering, Michael Okpara University of Agriculture, Umudike, Nigeria

**Keywords:** Engineering, Materials science

## Abstract

Due to the high costs of traditional concrete materials in Nigeria, such as river sand, there is an increasing demand to explore alternative materials like laterite for fine aggregates. Although laterite is abundant in Nigeria, its full potential in the construction industry remains untapped. Previous studies have shown that partially replacing river sand with laterite produces concrete with competitive strength properties. This research aims to validate and extend these findings, evaluating the impact of different aggregate sizes (12 mm, 20 mm, and 40 mm) on the strength of concrete with 10% and 25% laterite replacements for fine aggregate. Results revealed that as the laterite percentage increased, compressive, flexural, and split tensile strengths decreased. While 0% and 10% laterite replacements met the required strength, the mix with 25% laterite fell short. Increasing maximum coarse aggregate size led to higher strengths, with 40 mm sizes exhibiting the highest, and 12 mm the lowest. Compressive strengths ranged from 22.1 to 37.6 N/mm^2^, flexural strengths from 4.07 to 5.99 N/mm^2^ and split-tensile strengths from 2.93 to 4.30 N/mm^2^. This research highlights the need for meticulous mix design adjustments when using laterite, balancing workability with strength objectives. The developed regression models offer a valuable tool for predicting concrete properties based on mix parameters, providing insights for optimizing laterized concrete designs across diverse construction applications and supporting sustainable building practices.

## Introduction

Concrete, as a ubiquitous construction material, has undergone numerous innovations and adaptations to meet the evolving demands of modern construction practices that enhance the performance, sustainability, and resilience of built structures^1^. One such innovation is laterized concrete, a composite material that integrates the strength of traditional concrete with the distinctive properties of locally available laterite soil^2^. This amalgamation not only offers a sustainable approach to construction but capitalizes on the inherent advantages of laterite-rich regions, it also presents an opportunity to address environmental concerns and resource scarcity while redefining concrete's potential for structural applications. At the heart of this innovation lies the composition of aggregates, a fundamental component that significantly influences the mechanical and durability characteristics of the concrete matrix^3,4^.

The construction industry heavily relies on concrete as a fundamental building material due to its versatility, strength, and durability^5^. Nevertheless, concrete production demands extensive amounts of aggregates, predominantly comprising sand, gravel, and crushed stone^6^. These resources are limited, and excessive exploitation can exhaust them, causing scarcity and exacerbating environmental consequences^7^. Additionally, the process can result in soil erosion, water pollution, and disturbances to local ecosystems. Aggregates, comprising crushed stone, gravel, sand, and other granular materials, contribute to the overall strength, workability, and performance of concrete^8^. In the realm of laterized concrete, where the interplay between traditional aggregates and laterite soil is pivotal, the choice of aggregate size emerges as a critical parameter. The interaction between different aggregate sizes and laterite components yields a complex synergy that shapes the concrete's structural integrity, workability, and durability^9^.

The influence of aggregate sizes on the structural properties of conventional concrete has been extensively studied, yielding valuable insights into concrete's behavior under varying load conditions. The investigation into the effects of aggregate sizes on the structural properties of laterized concrete has gained attention as researchers and practitioners strive to optimize the performance of this innovative building material^10,11^. Nonetheless, the emergence of laterized concrete as an eco-friendly alternative has prompted the exploration of aggregate size effects in this unique material. Several studies have examined the effects of aggregate sizes on the mechanical properties of conventional concrete. However, limited research has specifically addressed these effects in laterized concrete^11,12^. A study by Salau and Busari^13^ investigated the compressive strength of laterized concrete with varying aggregate sizes. Their findings suggested that larger aggregates contributed to higher compressive strengths due to improved interlocking between the particles. Contrary to this, Raja and Vijayan^14^ found that smaller aggregates yielded higher compressive strengths in laterized concrete, attributing this to increased particle packing.

Also, Wei et al.^15^ investigated how different aggregate sizes influenced the mechanical properties of lightweight concrete. They found that the absence of medium-sized particles negatively affected density and compressive strength. Using a model, they estimated compressive strength, achieving up to 95 MPa after 90 days. Specimens with a single aggregate size showed weaker splitting tensile and flexural strengths compared to those with three sizes.

Moreover, Mohammed and Mahmood^16^, examined how the maximum aggregate size (MAS) in concrete, ranging from 12.5 to 50.0 mm, influenced ultrasonic pulse velocity (UPV). Concrete specimens were prepared with varying sand to aggregate ratios, water-cement ratios, and cement contents. UPV increased with larger MAS, and the study established relationships between UPV and compressive strength and UPV and Young's modulus for different MAS. Despite the potential benefits of laterized concrete, there is limited research addressing the effects of aggregate sizes on its structural properties. The existing body of knowledge primarily focuses on traditional concrete mixtures, and the applicability of those findings to laterized concrete is not straight forward. As such, there is a research gap that hinders our ability to design and construct laterized concrete structures with confidence and efficiency^17,18^. The identified gaps in the existing literatures include a limited focus on laterized concrete, insufficient exploration of its mechanical properties in relation to aggregate sizes, inadequate investigation of sustainable alternatives, a lack of standardized guidelines for aggregate selection, and the need for the integration of modern analytical techniques. Addressing these gaps would contribute to a more comprehensive understanding of laterized concrete's behavior and enhance sustainable construction practices.

This research endeavors to delve into the profound effects of aggregate sizes on the structural properties of laterized concrete. By conducting a systematic analysis of the influence of various aggregate sizes, we aim to unravel the intricate relationships between these components and shed light on the optimal aggregate size for enhancing the performance of laterized concrete in diverse engineering applications^19–21^. The findings of this research hold immense significance for the construction industry, academia, and sustainable development. By unraveling the intricate relationship between aggregate sizes and the structural properties of laterized concrete, this study contributes to the advancement of knowledge in sustainable construction materials. The insights gained from this research can guide engineers, architects, and policymakers in making informed decisions regarding the selection of aggregate sizes for laterized concrete, thereby enhancing the efficiency, safety, and durability of infrastructure projects. Furthermore, through the investigation of the impact of aggregate sizes on laterized concrete, this study contributes to the ongoing discourse on sustainable construction materials, aligning with global efforts to create environmentally responsible and resilient built environments.

## Methodology

### Materials

#### Fine aggregate

Fine aggregate, often referred to as sand, is an essential component of concrete. It provides cohesiveness to the mixture, fills the voids between larger particles, and contributes to the workability and strength of the concrete. The fine aggregates used for this research were sourced from the Calabar river and categorized as sharp sand passing through 2.36 mm sieve in accordance with the BS EN12620 standard.

#### Coarse aggregate

Coarse aggregate consists of larger particles such as gravel or crushed stone. It adds strength and stability to the concrete mix, enhancing its mechanical properties. The selection of appropriate sizes and types of coarse aggregate influences the structural characteristics of laterite concrete and conformed to BS EN12620. The coarse aggregates will be sourced from the Saturn quarry located in the Akamkpa Local Government Area of Cross River State, with a grain size ranging from 12 to 20 mm.

#### Cement

Cement is the binding agent that holds the concrete mixture together. The type and quantity of cement used affect the strength, durability, and setting time of laterite concrete. The cement employed in this experimental study is of the UNICEM brand, possessing a grade of 42.5 and conforms to BS EN 197–1:2011 specifications. It was procured from a materials market in Cross River State, Nigeria, and adhering to the specifications outlined in the Nigerian Industrial Standard (NIS) 444–1^22^.

#### Laterite

Laterite, a naturally occurring material, is a key ingredient in laterite concrete. It is a weathered clay-rich material that varies in color from reddish-brown to yellow obtained from Cross River State. When used in concrete, laterite serve as a partial replacement for fine aggregates, contributing to the sustainability and cost-effectiveness of the mixture^8^.

#### Water

Water is essential for the chemical reaction between cement and other components, allowing the concrete to set and gain strength. Potable water was used for this study in accordance with ASTM C1602-12 requirements.

### Experimental program

In this experiment, the concrete mix is modified by substituting fine aggregate with laterite across a range of 0% to 25% in line with relevant literature studies^21,23^. The target strength is set at 25 N/mm^2^, and the fine aggregate contents are varied to 518, 589, and 713 kg/m^3^. Additionally, the coarse aggregate contents are varied to 1027, 1251, and 1332 kg/m^3^, with cement contents of 380, 420, and 460 kg/m^3^, all while maintaining a water-cement ratio of 0.5. Furthermore, different coarse aggregate sizes of 12, 20, and 40 mm are employed. To conduct the experiment, the concrete ingredients are mixed thoroughly with water to ensure uniformity before being compacted and placed into molds for assessing mechanical strength taken the computed design mix parameters shown in Table 1. Freshly mixed concrete is also subjected to tests to determine its setting time and workability. Subsequently, the concrete samples are cured in a controlled environment at room temperature for a duration of 28 days. After this curing period, the samples are subjected to investigations aimed at assessing their structural properties^24,25^.

**Table 1:** Design mixes.

Sample names	Max. coarse agg. size (mm) @%laterite	Cement (Kg/m^3^)	Fine agg. river sand (Kg/m^3^)	Laterite soil (Kg/m^3^)	Coarse agg. granites (Kg/m^3^)	Water (Kg/m^3^)	Water-cement ratio
A1	12 @ 0%	460	713	0	1027	230	0.5
A2	20 @ 0%	420	589	0	1251	210	0.5
A3	40 @ 0%	380	518	0	1332	190	0.5
B1	12 @ 10%	460	641.7	71.3	1027	230	0.5
B2	20 @ 10%	420	530.1	58.9	1251	210	0.5
B3	40 @ 10%	380	466.2	51.8	1332	190	0.5
C1	12 @ 25%	460	534.75	178.25	1027	276	0.6
C2	20 @ 25%	420	441.75	147.25	1251	252	0.6
C3	40 @ 25%	380	388.5	129.5	1332	228	0.6

The mix ratio for the various grades of materials based on 25 N/mm^2^ mix design is given as 12 mm max size = 1:1.5:2.5; 20 mm max size = 1:1.5:3.0; 40 mm max size = 1:1.5:3.7.

### Methods

#### Aggregate impact value (AIV) test

The Aggregate Impact Value (AIV) test is a common procedure used to assess the resistance of aggregates to sudden impact or shock, reflecting their durability and toughness in construction applications. The test involves subjecting a sample of aggregates to impact using a specialized testing machine in accordance with (BS 812-112). The broken material is then collected, and its proportion is determined through sieve analysis. The Aggregate Impact Value is calculated, indicating the material's susceptibility to breakage under impact. A lower AIV suggests stronger aggregates, while a higher value indicates weaker ones. This test aids in evaluating the suitability of aggregates for various construction uses and ensures their quality and performance^26^. Calculate the Aggregate Impact Value using the formula in Eq. (1).

1 $${\text{AIV}} = \left[ {\left( {\text{Weight of broken material/Weight of original sample}} \right)} \right] \times 100$$

#### Aggregate crushing value (ACV) test

The Aggregate Crushing Value (ACV) test is a crucial procedure to determine the resistance of aggregates to crushing under gradually applied compressive loads. In subjecting a sample of aggregates to controlled pressure, the test evaluates their strength and durability, making it an essential factor in assessing their suitability for construction purposes. The ACV test involves applying increasing loads until the aggregates break, recording the maximum load applied, and then calculating the ACV in consonance with BS 812-110:1990 specification. A lower ACV indicates stronger aggregates, while a higher value suggests weaker ones prone to crushing. This test aids in classifying aggregates for various construction uses and ensures the quality and performance of materials^27^.

#### Flakiness index test

The Flakiness Index test is performed to assess the presence of flat and elongated particles in aggregates, which can impact the quality of construction materials. The procedure involves using a flakiness gauge to determine whether individual particles pass through a specified slot without turning in accordance with ASTM 4791-10 requirements. Through the calculation of the Flakiness Index, which represents the percentage of flat particles, the test provides insights into aggregate shape and angularity. A higher Flakiness Index indicates a greater proportion of undesirable particles, potentially affecting concrete performance. This test aids in ensuring the quality and suitability of aggregates for construction applications. The Flakiness Index is given by the formula in Eq. (2).

2 $$\left(\frac{{\text{Weight}} \, \mathrm{ of} \, \mathrm{ Sample}}{Weight \, of \, Flaky \, Sample}\right)\times 100$$

#### Compressive strength test

**Figure 1:**
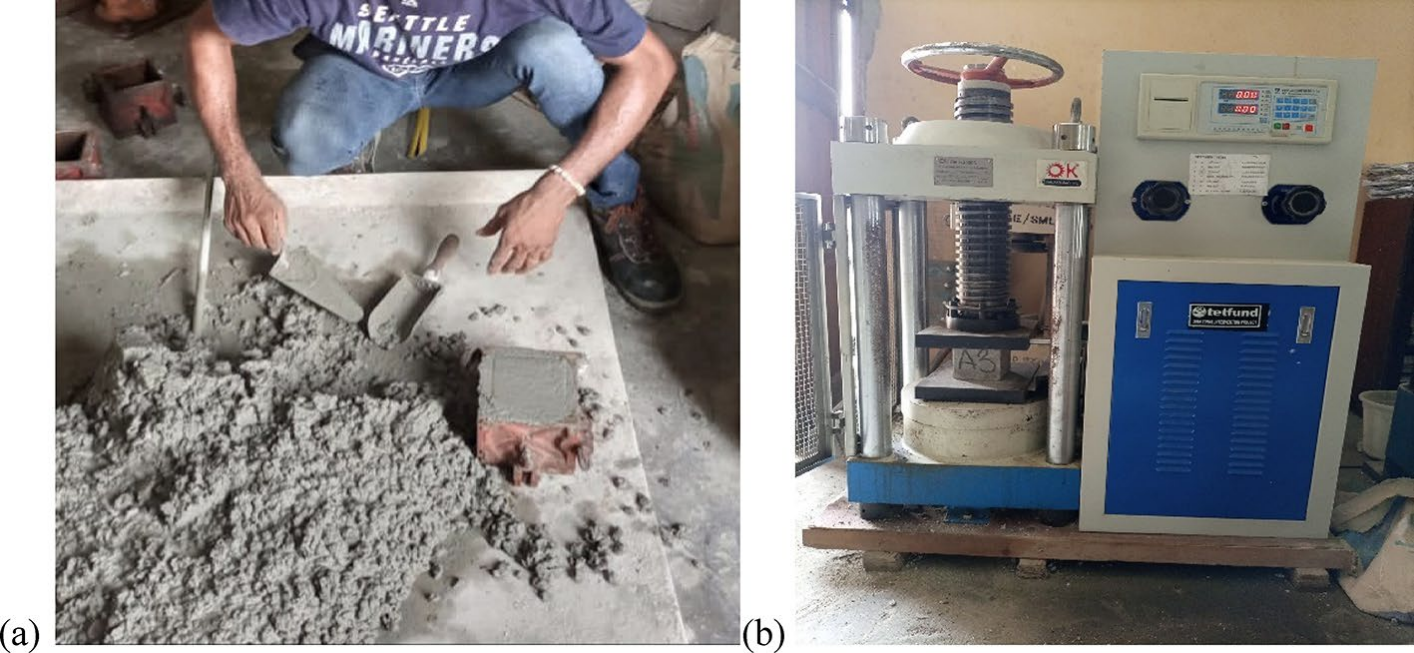
(**a**) Mixing and filling the cubic mold, (**b**) cube testing for compressive strength.

The compressive strength test assesses a material's capacity to endure axial loads without collapsing, a vital aspect for evaluating its structural behavior. This assessment holds significant value in appraising the concrete's quality and durability across varied construction applications. The materials employed in this investigation encompass cement, fine aggregates, laterite, coarse aggregates (crushed granite), and water in consonance with BS EN 12390-3:2019 provisions. The process involves preparing cubic molds measuring 100 × 100 × 100 mm for the nine specified experimental runs detailed in Table 1, with three replicates for each run. The test experimental apparatus and mixing procedure are shown in Fig. 1. A total of 108 laterized concrete samples were generated for the experiment, which were subsequently immersed in a curing tank for durations of 3, 7, 14, and 28 days. Following the hydration period, the concrete specimens' strength was evaluated by subjecting them to gradually applied axial load until reaching failure. The maximum applied load and specimen dimensions are used to compute the compressive strength^28,29^.

#### Flexural strength test

**Figure 2:**
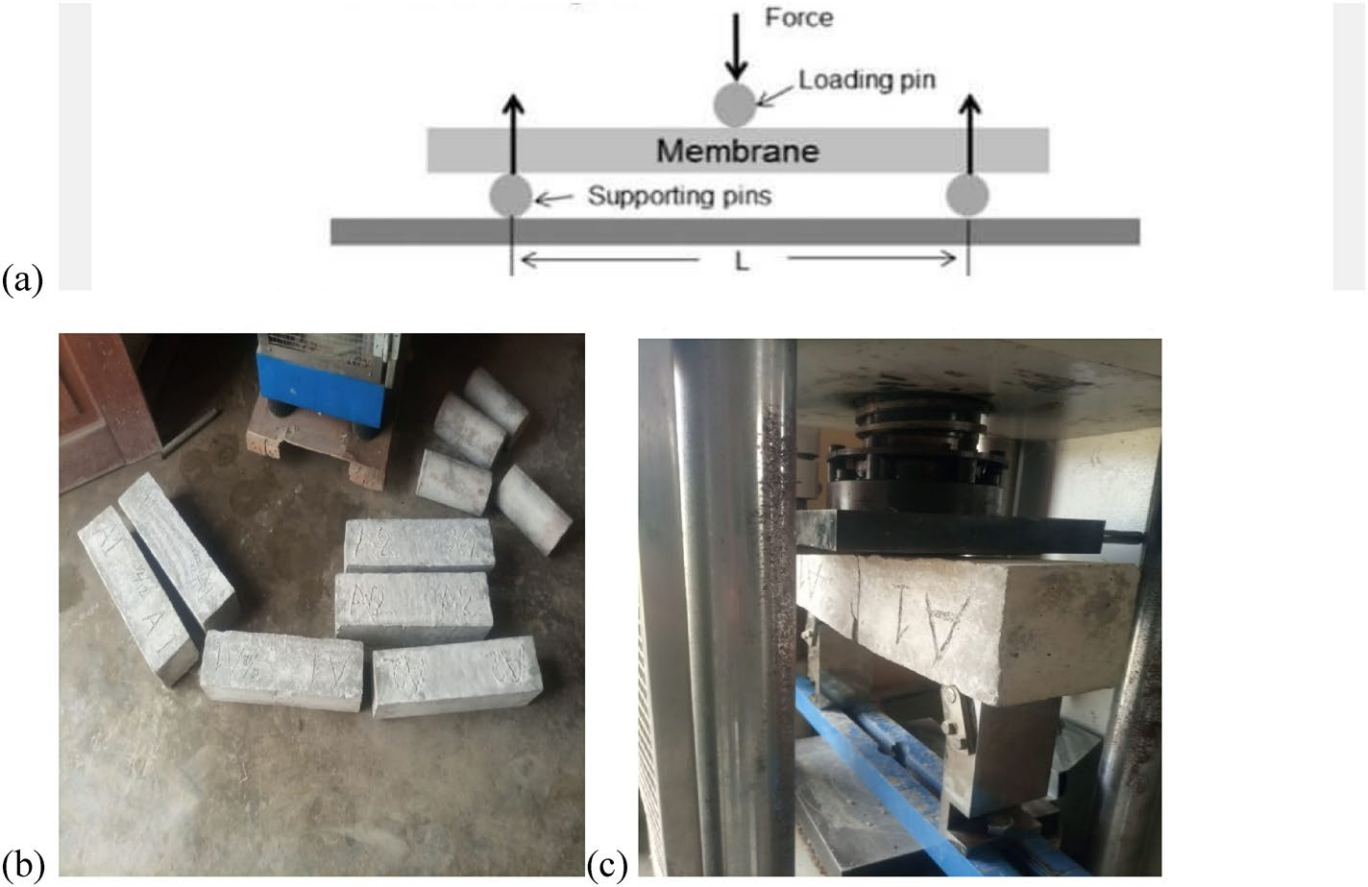
(**a**) Flexural strength test setup with the test sample for three-point test, (**b**) beams ready for testing, (**c**) beam testing by three-point test method.

Flexural Strength, also known as Modulus of Rupture, signifies the force a material can endure before fracturing or undergoing permanent deformation. It represents a material attribute, defined as the maximum stress a material withstands just prior to bending-induced yielding. The flexural test evaluates an unreinforced concrete beam or slab's capacity to resist bending-induced failure. Employing the same mixture proportions as mentioned earlier, each batch of concrete mix was employed to create three concrete beams (measuring 100 × 100 × 400 mm), subject to a 28-day curing period. Consequently, a total of 27 Concrete Beams were generated for assessing Flexural Strength during testing. During the designated timeframes after hydration, the Three-Point Test technique, also referred to as the central-point loading system, was employed to ascertain the Flexural Strength of distinct rectangular beam specimens in accordance with BS EN 12390-5:2019 requirement as shown in Fig. 2. In this approach, the rectangular beam sample is positioned atop roller supports on the testing apparatus, with 100 mm reference points from both beam ends and a 200 mm separation between the supports. These rollers establish a condition of simple support for the evaluation. Subsequently, a load is progressively applied to the midsection of the beam until the material undergoes rupture^30,31^.

#### Split tensile strength test

**Figure 3:**
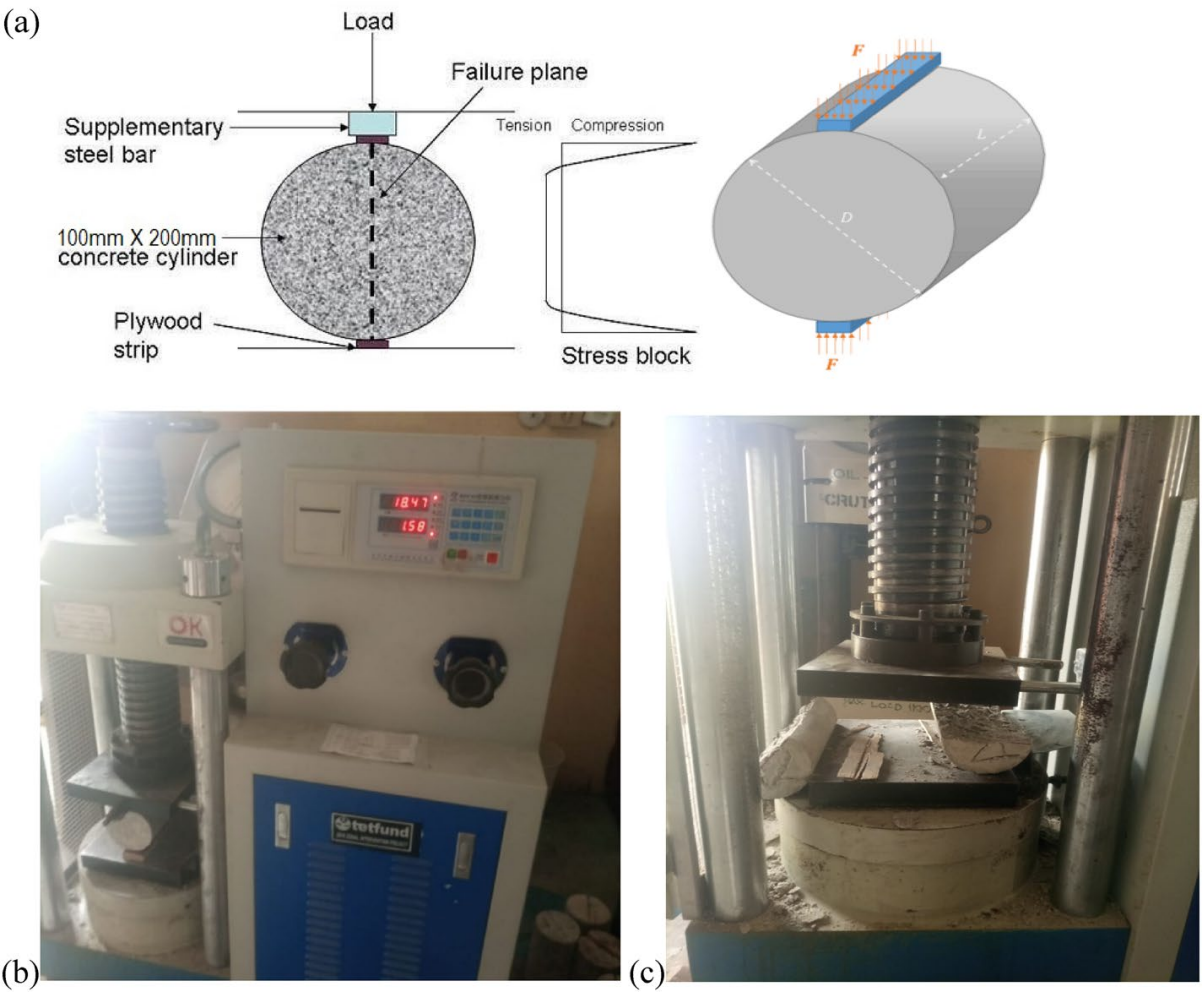
(**a**) Splitting tensile strength test of concrete. (**b**) cylinder sample under test, (**c**) the splitting of the cylinder sample.

This technique involves determining concrete's tensile strength using a cylindrical specimen that fractures along the vertical diameter. It's an indirect way to assess tensile strength. This method encompasses evaluating the splitting tensile strength of cylindrical concrete specimens, including molded cylinders. This property aids in designing lightweight concrete elements by gauging concrete's shear resistance. The test procedure involves subjecting cylindrical specimens to axial loading until they split along their length. The maximum load applied, and the specimen's dimensions are used to calculate the split tensile strength. The results of this test provide valuable data for assessing the concrete's ability to resist cracking and tension forces. Similar to the Flexural strength test, every concrete mix batch is used to create three cylindrical specimens (measuring 100 mm diameter and 200 mm height) which totals 27 concrete cylindrical samples, cured for 28 days following the guidelines defined in BS EN 12390-6:2019 as presented in Fig. 3^32^.

### Water absorption test

The water absorption test for concrete assesses the material's ability to absorb water and is crucial for evaluating its durability and permeability. The procedure involves weighing clean concrete specimens, immersing them in water, allowing them to soak for 24 h, and then determining their weight after surface drying. The water absorption percentage is calculated using the formula in Eq. (3), with higher values indicating increased porosity and reduced resistance to water penetration. Where W_1_ is the mass of each specimen to determine its initial dry weight and W_2_ Weigh the mass of the specimen after immersion to determine its saturated surface-dry weight. This test provides valuable insights into concrete's durability, especially in construction applications where resistance to water ingress is essential^33^.

3 $${\text{Water}} \, \mathrm{ Absorption }(\mathrm{\%}) =\frac{{W}_{2}-{W}_{1}}{{W}_{1}}\times 100$$

### Data analysis techniques

Data analysis is a crucial process that involves inspecting, cleaning, transforming, and interpreting data to uncover valuable insights, patterns, and trends. It enables researchers, analysts, and decision-makers to draw meaningful conclusions, make informed decisions, and solve complex problems such as evaluating the mechanical strength of laterized concrete samples. Data analysis techniques encompass a wide range of methods, including descriptive statistics, visualization, regression analysis, hypothesis testing, and more. These techniques allow researchers and engineers to extract meaningful insights from the collected data regarding the concrete's mechanical properties^34,35^.

#### Descriptive statistics

Descriptive statistics are fundamental tools used to summarize and describe key characteristics of a dataset. They provide a clear snapshot of data by offering measures of central tendency (mean, median, mode) that indicate the typical value, and measures of variability (standard deviation, range, interquartile range) that depict how spread out the data points are. Descriptive statistics aid in understanding the data's distribution, identifying outliers, and making initial insights without delving into complex analyses. They are essential for researchers, analysts, and decision-makers seeking to grasp the basic nature of their data before proceeding to more advanced statistical analyses. These statistics help researchers understand the distribution and variability of mechanical strength values in the concrete samples^36,37^.

#### Scatter plots and correlation analysis

Scatter plots and correlation analysis are tools used in statistics to explore the relationship between two variables. A scatter plot is a graphical representation of data points, where each point represents a pair of values from the two variables. Scatter plots help visually assess the direction and strength of the relationship between variables, assess the strength of relationships, and make data-driven decisions^38^. Correlation analysis quantifies the degree and direction of the linear relationship between two variables. The correlation coefficient, ranges from − 1 to + 1. A positive value indicates a positive correlation (as one variable increases, the other tends to increase), a negative value indicates a negative correlation (as one variable increases, the other tends to decrease), and a value close to zero suggests a weak or no linear correlation. Scatter plots can be used to examine the relationship between different variables such as ingredients mixture combinations, aggregates sizes, curing durations and mechanical strength^39^.

#### Regression analysis

Regression analysis is a statistical technique used to understand the relationship between a dependent variable and one or more independent variables. It aims to find a mathematical equation that best describes the connection between these variables. The result is a regression model that can be used for prediction, hypothesis testing, and understanding how changes in the independent variables affect the dependent variable. There are various types of regression analysis, including linear regression (for linear relationships), multiple regression (for multiple independent variables), and nonlinear regression (for curved or nonlinear relationships)^40^. Regression analysis helps establish predictive models that relate mechanical strength to various influencing factors. By fitting regression models, researchers can quantify the impact of individual variables on mechanical strength and predict strengths based on given conditions. Regression analysis involves developing mathematical equations that describe the relationship between variables^41,42^. Here are the equations for simple linear regression and multiple linear regression:

##### Simple linear regression

In simple linear regression, we have one dependent variable (Y) and one independent variable (X). The relationship is represented by a straight-line formula shown in Eq. (1):

$$Y= {\beta }_{0}+ {\beta }_{1}x+\varepsilon$$

where *Y* is the dependent variable (response); *x* is the independent variable (predictor); ${\beta }_{0}$ is the y-intercept; ${\beta }_{1}$ is the slope of the line; *ε* represents the error term (unexplained variation)^43^.

##### Multiple linear regression

In multiple linear regression, we have multiple independent variables (X_1_, X_2_, …, X_k_) that can influence the dependent variable (Y). The relationship is represented by a linear equation:

$$Y= {\beta }_{0}+ {\beta }_{1}{x}_{1}+{\beta }_{2}{x}_{2}+\dots +{\beta }_{k}{x}_{k}+\varepsilon$$

where *Y* is the dependent variable; *x*_1_, *x*_2_,…, *x*_*k*_ are the independent variables; ${\beta }_{0}$ is the intercept; ${\beta }_{1, } {\beta }_{2 },\dots , {\beta }_{k}$ are the coefficients for the independent variables; *ε* represents the error term.

These equations serve as the foundation for regression analysis, where the goal is to estimate the values of the coefficients (${\beta }_{1, } {\beta }_{2 },\dots , {\beta }_{k}$) that best fit the observed data. Regression analysis allows us to quantify the relationship between variables, make predictions, and understand the impact of changes in the independent variables on the dependent variable^44^.

#### Statistical significance testing

Statistical significance testing is a critical analysis method used to determine if observed differences or relationships in data are likely due to actual effects or are simply the result of random variability. It involves comparing sample data to a null hypothesis, which assumes that there is no real effect or difference in the population^45^. Statistical significance testing helps researchers make informed decisions and draw conclusions based on data. Analysis of variance (ANOVA) statistical testing is adopted in this research study which assesses the variability within and between groups to determine if the observed differences in means are likely due to actual effects or random chance^46^. In testing the experimental results, ANOVA help to examine whether there are statistically significant differences in mechanical strength among different groups or conditions, such as aggregate size categories, factor levels and curing durations. ANOVA assesses the variability within and between groups to determine if the observed differences in means are likely due to actual effects or random chance^47^.

### Consent to participate

All authors were highly cooperative and involved in research activities and preparation of this article.

## Results discussion and analysis

Results discussion and analysis involves interpreting and understanding the outcomes of various tests conducted on the produced laterized concrete samples with different aggregate sizes. The results of the study on the effects of aggregate sizes on the structural properties of laterized concrete provide valuable insights into the behavior of this composite material. The analysis encompassed various aspects, including compressive strength, flexural strength, and splitting tensile strength, each shedding light on how the variation in aggregate sizes influences the overall performance of the concrete. This information aids in the informed design and construction of concrete structures, ensuring they meet both performance and safety requirements^48,49^.

### Test materials characterization

**Figure 4:**
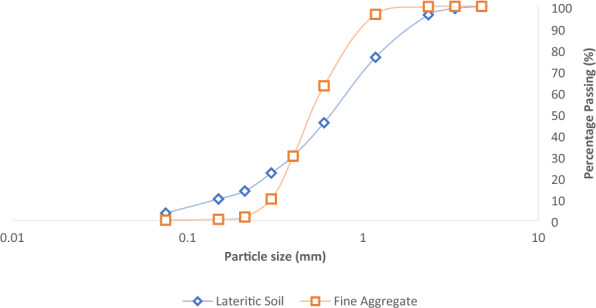
Particle size distribution curve for laterite soil.

Preliminary laboratory tests were conducted on the ingredient materials to assess their overall engineering behaviour for civil works in line with specified guidelines. In order to achieve this goal, the test coarse aggregates underwent aggregate impact value (AIV), aggregate crushing value (ACV), flakiness index and specific gravity tests while the coarse aggregates, river sand and laterite were tested for specific gravity^50^. More so, sieve analysis and test were carried out on the lateritic soil and river sand samples. The physical test results summary for the aggregates are shown in Table 2, the sieve analysis plot for the aggregate materials is presented in Fig. 4. The results for the coarse aggregates indicated AIV and ACV of 9.16% and 17.55 respectively which signified excellent quality aggregates with high resistance to impact and suitable for most construction purposes^51^. However, with a flakiness index value of 15% which implies presence of some flaky particles, but the overall shape and size distribution are suitable for various construction applications^52^. The gradation coefficients derived from particle size distribution results indicate that both river sand and laterite, serving as fine aggregates, exhibit well-graded characteristics with a relatively uniform particle size distribution. This suggests their suitability for a range of construction applications. In the case of lateritic samples, the results suggest moderately well-graded aggregates, maintaining acceptable gradation characteristics with a balanced particle size distribution^53^.

**Table 2:** Results for the physical properties of test materials.

Description	Laterite	River Sand	Coarse aggregate		
			12 mm	20 mm	40 mm
Specific gravity	2.68	2.66	2.93	2.92	2.76
Flakiness index			15%		
Aggregate impact value			9.16%		
Aggregate crushing value			17.55%		
Particle size distribution					
D_10_	0.158 mm	0.315 mm			
D_30_	0.395 mm	0.432 mm			
D_60_	0.873 mm	0.585 mm			
Coefficient of uniformity (C_u_)	6.8	1.857			
Coefficient of curvature (C_c_)	0.15	1.013			
Compaction					
Maximum dry density	1.874 g/cm^3^				
Optimum moisture content	10%

#### Chemical properties

Concrete consists of different components with unique chemical characteristics. Grasping the chemical properties of these materials is vital to evaluate how well they work together, potential reactions, and the durability of concrete structures over time. This knowledge is essential for minimizing negative impacts, fine-tuning the mixture, and ensuring the desired chemical behavior and overall performance^54^. The oxide composition of the materials, including fine sand, crushed granite, laterite, and cement, was determined through XRF testing, as depicted in Table 3. Laterite exhibits oxide properties quite like river sand in terms of clinker requirement^55^. Additionally, the magnesium oxide (MgO) content remains below 3%. Notably, laterite boasts a high SiO_2_ content of 54.02% and a significantly low CaO content. Interestingly, this aligns with the criteria for Natural Pozzolans, which stipulates that SiO_2_, Al2O_3_, and Fe_3_O_2_ should account for at least 70% of the total oxides according to ASTM C618, 98. It's also apparent that river sand, crushed granite, and laterite all feature comparable levels of silica (SiO_2_) content^56^. This oxide, silica, dominates the composition in all these materials and holds substantial importance in cement formulation. By fostering the creation of di-calcium silicate and tri-calcium silicate, silica bolsters the cement's strength. Nevertheless, excessive silica content can impede setting rates and therefore requires monitoring^57,58^.

**Table 3:** Oxides composition of materials.

Oxides	Fine sand%	Crushed granite%	Laterite%	Cement %
SiO_2_	76.77	67.52	54.02	26.65
AL_2_O_3_	8.91	12.63	22.34	17.22
Fe_2_O_3_	4.25	6.51	10.15	5.98
CaO	3.79	4.24	2.78	36.46
MgO	2.02	1.87	1.84	2.10
SO_3_	0.07	0.09	0.00	2.22
MnO	0.18	0.00	0.12	0.00
K_2_O	1.34	3.20	0.08	0.88
TiO_2_	0.18	0.00	0.51	0.36
Na_2_O	1.03	1.11	0.00	0.03
LOI	1.74	2.80	7.95	8.39
SiO_2_ + Al_2_O_3_ + Fe_3_O_2_	89.93	86.66	86.51	

### Workability of the laterized concrete

**Figure 5:**
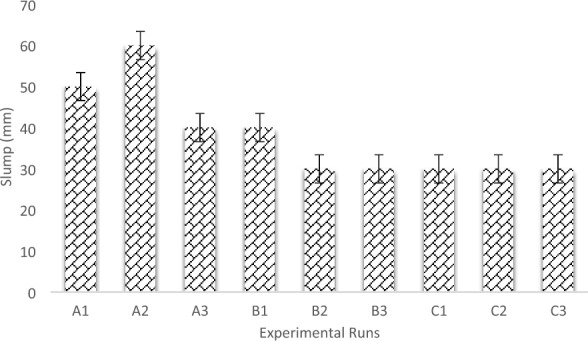
Water-cement ratio versus slump values.

Workability of laterized concrete refers to its ease of handling, placing, and compacting during construction. The presence of laterite as a replacement for fine aggregates can impact workability behavior due to their angular shape and water absorption. The laterite particles can increase friction, making the fresh concrete harder to move and compact^59^. Workability tests, such as the slump test, assess how easily the concrete can be placed and compacted. Achieving the right workability balance is essential for efficient construction and maintaining quality standards. The research's concrete production incorporated the Slump test to determine workability, with Fig. 5 illustrating the slump values for distinct concrete batches concerning the employed water-cement ratio and varying aggregate sizes. The obtained results show that workability reduces as the proportion of Laterite soil increases in the concrete mix^60,61^. This reinforced the findings of Falade^59^, who discovered from his study that the water requirement for a given concrete mixture increases with an increase in the proportion of laterite soil present in the mixture. This proves that the Laterite soil is more water-absorbent than the river sand. With a water-cement ratio of 0.5, the Control mix (concrete with 0% laterite) produced true slumps of 50 mm, 60 mm and 40 mm for batches A1, A2, and A3 respectively. Also, with a water-cement ratio of 0.5, a concrete mix with 10% fine aggregate of laterite produced true slumps of 40 mm, 30 mm, and 30 mm for concrete batches of B1, B2, and B3 respectively. However, to produce true slumps of 30 mm across concrete mixes of C1, C2, and C3 for 25% laterite soil content of the fine aggregate, the water-cement ratio increased to 0.6.

Moreover, the results obtained showed a reduction in setting time properties as the lateritic content increased in the concrete matrix. Simultaneously, an increase in aggregate sizes from 12 to 20 mm led to a maximum slump response ranging from 50 to 60 mm for the control concrete samples. However, a further increase in aggregate sizes to 40 mm resulted in a decrease in the slump response to 40 mm. Additionally, in the case of laterized concrete, the slump results tended to decrease with an increase in aggregate size for 10% laterite concrete. Conversely, the slump values remained constant as aggregate sizes increased for 25% lateritic concrete. This indicates that the incorporation of laterite and variations in aggregate size have noticeable effects on workability properties in the concrete mixtures. The results suggest a nuanced relationship between these factors, providing insights for optimizing concrete mix designs based on specific performance requirements^8^.

### Mechanical properties of laterized concrete

The mechanical properties of laterized concrete encompass its strength, durability, and overall structural behavior. These properties are influenced by the incorporation of laterite as a substitute for conventional aggregates. The mechanical properties of laterized concrete are determined through various tests, including compressive strength, flexural strength, splitting tensile strength.

#### Compressive strength test results

The compressive strength results of laterized concrete provide valuable insights into its load-bearing capacity and structural performance. Compressive strength is the maximum load a concrete specimen can withstand before experiencing failure under axial loading. For laterized concrete, which incorporates laterite as a replacement for conventional aggregates, the compressive strength results can reveal how this substitution influences the concrete's mechanical behavior^62^. The analysis of compressive strength results involves comparing them with design requirements and relevant standards. It helps engineers and professionals assess the concrete's suitability for different civil construction applications. The impact of varying proportions of laterite on compressive strength can guide mix design optimization for achieving desired strength levels while using alternative aggregates^63^.

**Figure 6:**
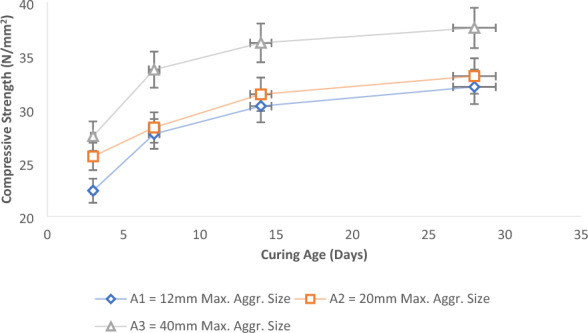
Effect of aggregate sizes on the compressive strength of control concrete.

The experimental results for the control samples without laterite materials known as Sample A (0% laterite, 100% River Sand, and each of 12 mm, 20 mm, and 40 mm maximum aggregate sizes), for the various curing ages is presented in Fig. 6. The findings indicated a direct relationship between the compressive strength of the concrete and the increase in the maximum coarse aggregate sizes. It was noted that the overall strength exhibited a linear increase as the hydration periods for the test concrete samples were extended^64^. This outcome aligns with previous studies conducted by Salau and Busari^13^ and Mohad and Saim^65^. In the case of the control mix with 100% river sand at 28 days and using varying maximum sizes of granite aggregates (12 mm, 20 mm, and 40 mm), the corresponding compressive strengths were determined as 32.1 N/mm^2^, 33.1 N/mm^2^, and 37.6 N/mm^2^, respectively. These results denote a progressive enhancement in strength: a 3% rise between the 12 and 20 mm samples, a 13.6% surge between the 20 and 40 mm samples, and a substantial 17% increase when comparing the 12 mm and 40 mm maximum aggregate samples. This signifies that a larger aggregate size contributes to increased concrete strength when utilizing a well-graded coarse aggregate. Interestingly, this contradicts certain studies suggesting that smaller aggregate sizes yield higher compressive strength compared to larger sizes^66^.

**Figure 7:**
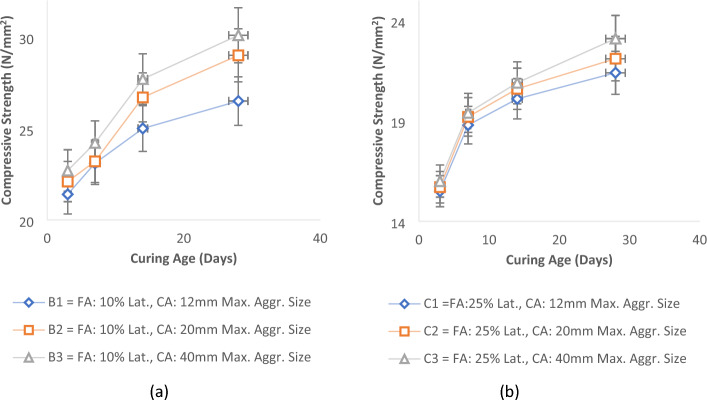
Effect of aggregate sizes on concrete’s strength for (**a**) 10% of laterite in fine aggregate and (**b**) 25% laterite in fine aggregate.

The same trend is evident in cases involving samples with partial replacement of fine aggregate by laterite soil. This applies to Sample B, which incorporates 10% laterite soil in lieu of river sand, and Sample C, featuring a 25% replacement of river sand with laterite soil as delineated in Fig. 7a,b. The results showed that alterations in the coarse aggregate sizes similarly yielded variations in the compressive strength of the concrete. Comparable to the scenario with normal concrete (Sample A), an increase in aggregate sizes corresponded to an upsurge in concrete strength. Nevertheless, the magnitude of these changes was comparatively less pronounced than those observed in normal concrete. The provided illustrations reveal that with an escalating proportion of laterite soil in the fine aggregate, the influence of aggregate sizes became less significant. Nevertheless, the consistent pattern of larger aggregate sizes contributing to enhanced compressive strength persisted across all sample batches. This is in consonance with the research findings of Saravanan et al*.*^67^.

#### Effect of the proportion of laterite soil on the compressive strength of concrete

The effect of the proportion of laterite soil on the compressive strength of concrete is a crucial aspect of concrete mix design. As the content of laterite soil in the fine aggregate increases, it tends to influence the overall behavior of the concrete. This influence is particularly notable in terms of compressive strength, which is a key indicator of concrete's load-bearing capacity^68^. The effect of the proportion of laterite soil on the compressive strength of concrete underscores the importance of accurate mix design and understanding the interactions between different materials in the concrete matrix. Balancing the desired characteristics of the concrete with the impact on compressive strength is a crucial aspect of producing durable and reliable concrete structures^69^.

**Figure 8:**
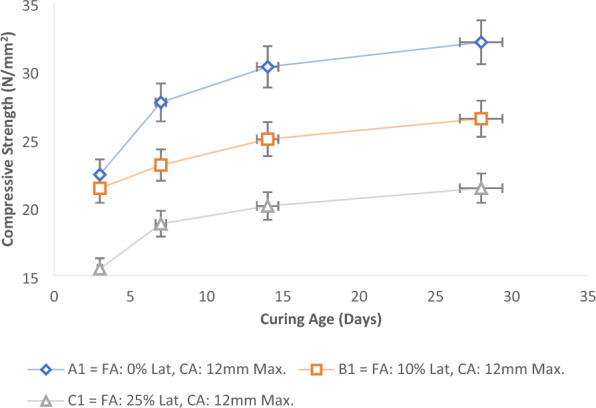
Effect of laterite soil ratios on concrete’s strength with 12 mm max. size of coarse aggregate.

**Figure 9:**
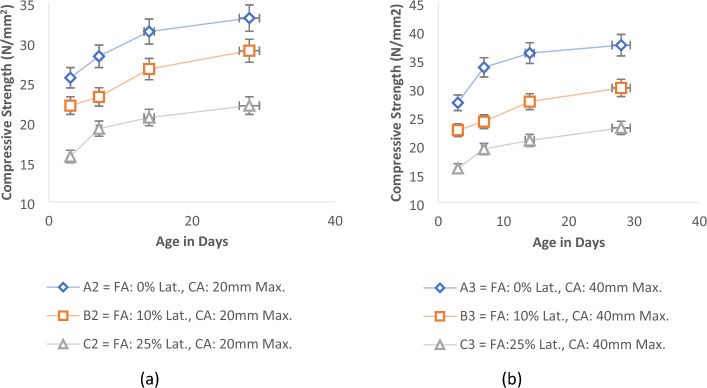
Effect of laterite soil ratios on concrete’s strength for (**a**) 20 mm max. size of coarse aggregate and (**b**) 40 mm max. size of coarse aggregate.

Figure 8 illustrates the impact of different proportions of laterite soil on the strength of concrete. In this investigation, we employed 0%, 10%, and 25% partial replacements of fine aggregate with laterite soil. A similar proportion of laterite was also tested with 20 mm and 40 mm maximum coarse aggregate sizes, yielding comparable outcomes shown in Fig. 9a,b. Notably, due to the variation in aggregate sizes, the compressive strengths exceeded those of the 12 mm aggregate size.

The graphical results highlight substantial strength disparities between control mixes and concrete mixes containing varying proportions of laterite soil. As the percentage of laterite soil in the mix increased, concrete's compressive strength decreased. A prudent choice seems to be a 10% replacement of fine aggregate with laterite soil, yielding a compressive strength of 30.1 N/mm^2^ at 28 days, surpassing the specified characteristic strength (C25). Similarly, a 25% laterite proportion in fine aggregate with a 40 mm maximum aggregate size produced a compressive strength of 23.1 N/mm^2^, slightly below the specified characteristic of 25 N/mm^2^, yet effective. With a 20 mm maximum coarse aggregate size, 25% laterite soil in fine aggregate yielded a compressive strength of 22.1 N/mm^2^, aligning with the findings of Salau and Busari^13^, and Ata^70^.

Overall, all aggregate size grades (12 mm, 20 mm, and 40 mm) with a 10% laterite proportion in fine aggregate yielded compressive strengths surpassing 25 N/mm^2^ at 28 days, with a density exceeding 2000 kg/m^3^. Meanwhile, a 25% laterite proportion in fine aggregate produced compressive strengths surpassing 20 N/mm^2^, maintaining a density above 2000 kg/m^3^, albeit lower than the specified characteristic strength of 25 N/mm^2^. Additionally, compressive strength increased as concrete age advanced across all scenarios. Depending on the intended application, it is advisable to consider a partial replacement of fine aggregate with up to 20% laterite soil, particularly when the laterite soil shares properties similar to those used in this study.

#### Flexural strength test results

**Figure 10:**
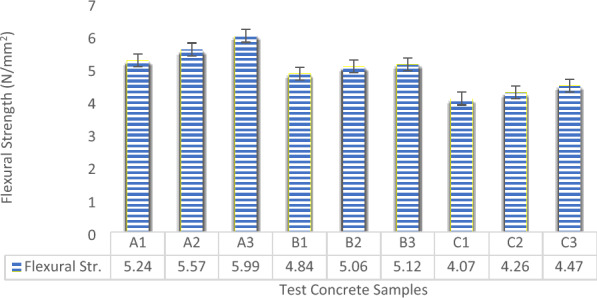
Flexural strength results.

The Flexural Strength Test examines how well a material withstands bending forces. In the case of laterized concrete, this test evaluates its ability to resist bending stresses without fracturing. The study incorporated various proportions of laterite soil as a partial replacement for fine aggregate. The Flexural Strength test of the experimental concrete was determined by the creation of concrete beams of dimensions 100 mm × 100 mm × 400 mm for the various batches of the concrete produced and cured for 28 days^71^. These results emphasize the intricate interplay between laterite soil proportion, aggregate size, and flexural strength in laterized concrete as shown in Fig. 10. The graphical outcomes revealed that within the set of samples labeled as “A,” sample A3 exhibited the highest flexural strength, followed by sample A2, while sample A1 demonstrated the lowest flexural strength. This pattern implies that, similar to the trend observed in compressive strength, the flexural strength increased as the maximum coarse aggregate sizes were raised. Furthermore, when evaluating samples A, B, and C—representing 0%, 10%, and 25% replacement of river sand with laterite respectively—it became evident that as the percentage of laterite in the concrete increased, the flexural strength decreased. These experimental findings underscore the imperative of precise adjustments to these variables to attain the desired flexural characteristics, customized for specific construction demands in line with the findings of Ndububa and Osadebe^72^.

#### Split tensile strength test

**Figure 11:**
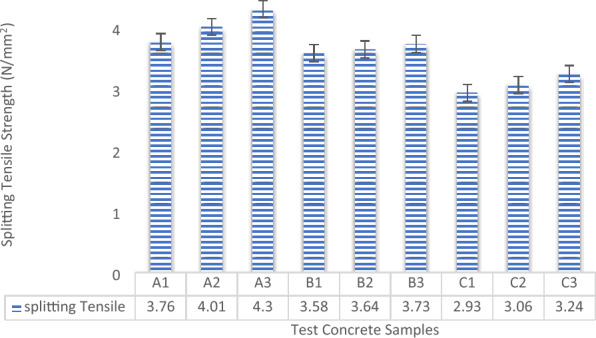
Splitting tensile strength results.

The split tensile strength test evaluates the tensile strength of concrete by inducing a splitting failure along its diameter. This test provides valuable insights into the material's tensile properties and behavior, especially in relation to cracking and durability. In the context of laterized concrete, this test helps determine how the inclusion of laterite affects the concrete's tensile strength. The split tensile strength results of laterized concrete offer insights into how the varying proportions of laterite and other constituents impact the material's performance under tensile stresses^73^. The presented graph illustrates the splitting tensile strength results of the experimental concrete samples that underwent a 28-day curing period. This assessment aimed to analyze the strength performance concerning the aggregate materials' proportions within the concrete mixture, as depicted in Fig. 11. Observations from the outcomes indicate that the splitting tensile strength of the tested concrete increased as the maximum aggregate sizes grew larger. Consequently, the concrete incorporating 40 mm coarse aggregate exhibited notably higher splitting tensile strength across all produced samples, reaching a maximum of 4.3 N/mm^2^ for the control sample with 40 mm coarse aggregate. Conversely, concrete with 12 mm coarse aggregate displayed the lowest splitting tensile strength among all mixes, measuring 2.93 N/mm^2^ for the concrete with 0% laterite (control). These results, aligning with the outcomes reported by Oguaghamba and Onyia^74^, offer valuable insights into the suitability of laterized concrete for various construction purposes. Additionally, they contribute to the enhancement of mix proportions to attain specific structural attributes.

#### Water absorption test

The laterized concrete specimens were prepared accordingly and initial weights taken for cube samples hydrated for 7-, 14- and 28-days period for 0%, 10% and 25% lateritic content with respect to maximum aggregate sizes of 20 mm and 40 mm respectively. The samples were further immersed in water for 24 h and weighed to determine the absorption rate using Eq. (3) and the computed results are shown in Table 4. The results showed that as the curing period increased, the water absorption of concrete samples with no laterite content (0%) also increased. In contrast, concrete containing 10–25% lateritic material exhibited the lowest water absorption after 14 days of curing. Additionally, larger maximum aggregate sizes were associated with higher water absorption values in all concrete specimens. Overall, the water absorption tests indicated good permeability for structural applications. Conventional concrete had water absorption values ranging from 2.48 to 3.65%, while concrete with 10–25% lateritic content had values ranging from 3.13 to 4.75%. This suggests that the addition of laterite may slightly increase water absorption but still maintains reasonable permeability levels for structural purposes.

**Table 4:** Water absorption for specimens with 20–40 mm max aggregate sizes at different curing age.

Hydration period (days)	Water absorption (%)					
	0% laterite		10% laterite		25% laterite	
	20 mm Max size	40 mm Max size	20 mm Max size	40 mm Max size	20 mm Max size	40 mm Max size
7	2.48	2.81	3.29	3.65	3.82	4.44
14	2.75	2.92	3.13	3.56	3.79	4.31
28	3.35	3.65	3.68	3.82	4.07	4.75

### Analysis of data

**Figure 12:**
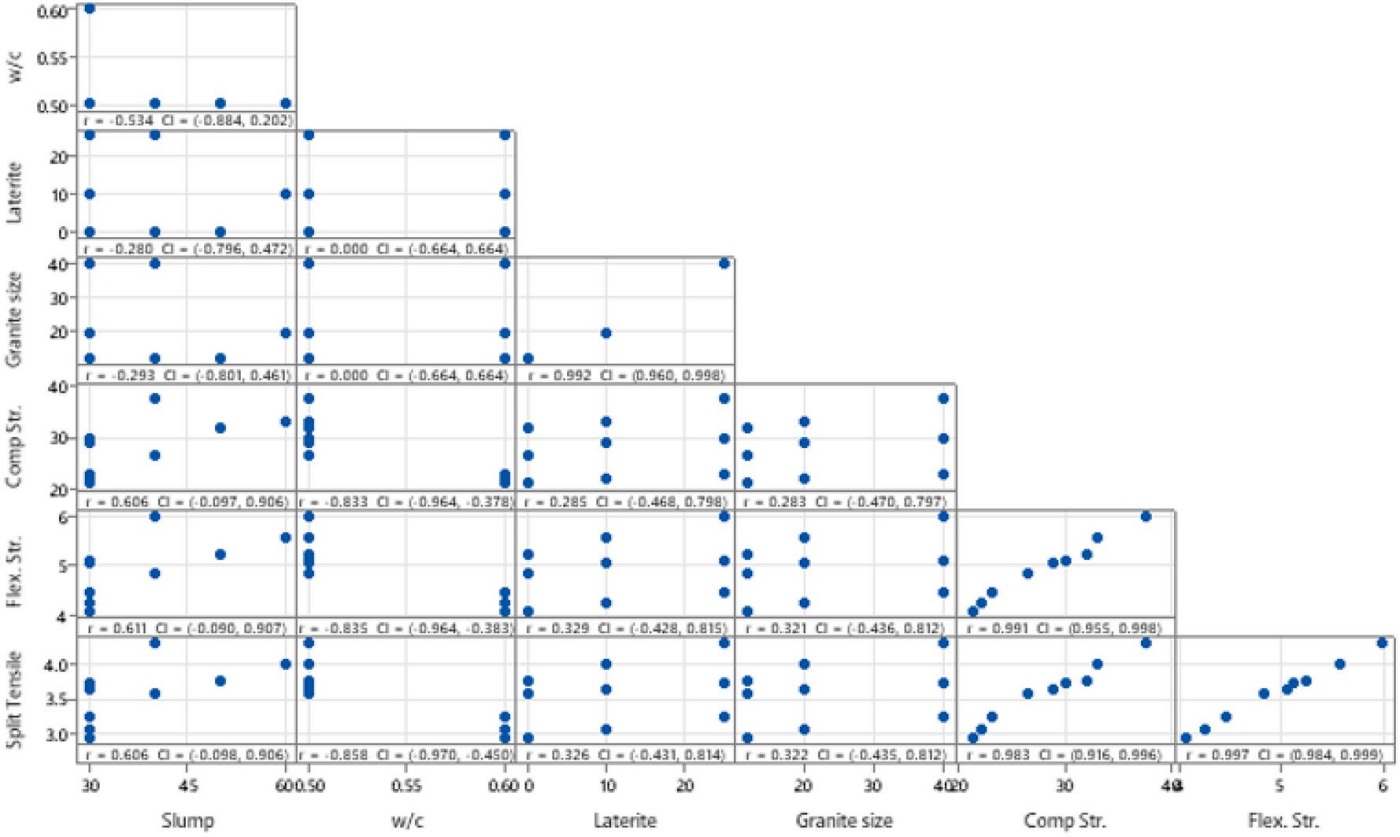
matrix correlation plot.

Statistical analysis of experimental data on the mechanical strength behavior of laterized concrete involves using statistical techniques to analyze and interpret the results obtained from testing the concrete samples. This analysis aims to uncover trends, relationships, and variations within the data, offering valuable understandings into the performance of the material^75^. The objective of this analysis is to reveal patterns, associations, and fluctuations present in the data, Statistical analysis helps researchers draw meaningful conclusions from experimental data, providing insights into the effects of different factors such as mixture proportions, slump and aggregate sizes on the mechanical strength behavior of laterized concrete and supporting evidence-based decision-making in construction and engineering projects^76,77^. The derived experimental results from the mechanical properties evaluation exercise of the laterized concrete with varying sizes of coarse aggregates were tabulated for analysis purposes as presented in Table 5. The analysis was carried out using Minitab 21 software to derive the correlation analysis and descriptive statistics of the datasets shown in Tables 6 and 7 and the matrix Pearson correlation graphical plot showing the confidence intervals (CI) in Fig. 12. The Matrix correlation plots are useful for quickly identifying relationships within the experimental dataset to pinpoint which variables are positively or negatively correlated, which can inform further analysis, model building. The results describe the main features of the dataset, helping to understand its central tendencies, variability, and distribution. The derived statistical results provide crucial insights into the data's characteristics and connections, facilitating informed decision-making and further analysis^78^. The computed statistical results indicated average maximum positive correlation of 0.99 between flexural and splitting tensile strength properties while maximum correlation of 0.992 was computed for laterite vs. granite independent variable. Conversely, maximum negative correlation of − 0.84 was recorded for compressive, flexural, and splitting tensile strength vs. water-cement ratio. This indicated that decrease in water-cement ratio resulted in increase in the strength response of the laterize concrete samples.

**Table 5:** Datasets for statistical evaluation.

Slump	w/c	Laterite	CA size	Comp str.	Flex. str.	Split tensile
50	0.5	0	12	32.1	5.24	3.76
60	0.5	10	20	33.1	5.57	4.01
40	0.5	25	40	37.6	5.99	4.3
40	0.5	0	12	26.5	4.84	3.58
30	0.5	10	20	29	5.06	3.64
30	0.5	25	40	30.1	5.12	3.73
30	0.6	0	12	21.4	4.07	2.93
30	0.6	10	20	22.1	4.26	3.06
30	0.6	25	40	23.1	4.47	3.24

**Table 6:** Correlation analysis results.

	Slump	w/c	Laterite	Granite size	Comp str.	Flex. Str.
w/c	− 0.534					
Laterite	− 0.280	0.000				
Granite size	− 0.293	0.000	0.992			
Comp Str	0.606	− 0.833	0.285	0.283		
Flex. Str	0.611	− 0.835	0.329	0.321	0.991	
Split Tensile	0.606	− 0.858	0.326	0.322	0.983	0.997

**Table 7:** Descriptive statistics results.

Variable	Mean	SE mean	Std. dev.	Variance	Minimum	Maximum	Range	Skewness	Kurtosis
Slump	37.78	3.64	10.93	119.44	30.00	60.00	30.00	1.29	0.77
w/c	0.5333	0.0167	0.0500	0.0025	0.5000	0.6000	0.1000	0.86	− 1.71
Laterite	11.67	3.63	10.90	118.75	0.00	25.00	25.00	0.29	− 1.71
Granite size	24.00	4.16	12.49	156.00	12.00	40.00	28.00	0.57	− 1.71
Comp Str	28.33	1.84	5.52	30.50	21.40	37.60	16.20	0.23	− 0.92
Flex. Str	4.958	0.207	0.621	0.385	4.070	5.990	1.920	0.13	− 0.61
Split Tensile	3.583	0.148	0.443	0.196	2.930	4.300	1.370	− 0.01	− 0.61

#### Regression analysis

Multiple linear Regression analysis of laterized concrete's mechanical properties results aims to uncover the mathematical relationships between the test experimental variables. It seeks to model how changes in independent variables (such as aggregate sizes, slump, w/c and proportions of laterite) influence dependent variables (like compressive strength, flexural strength, and splitting tensile strength). By fitting a regression equation to the data, it enables prediction, understanding trends, and identifying significant factors affecting concrete's mechanical properties. ANOVA was further deployed to assess the significance of variations among different factors on the observed outcomes in the experimental data^79^.

ANOVA helps determine if there are statistically significant differences in the means of mechanical properties (response) based on various factors like aggregate sizes, proportions of laterite, w/c and slump as shown in Tables 8, 9, and 10 for compressive strength, flexural strength and splitting tensile strength respectively. By partitioning the total variability into components attributed to different sources of variation, ANOVA helps to identify which factors have the most substantial impact on the concrete's mechanical strength performance^80,81^. The obtained computation results showed that there is statistical significance between the variables at a 95% confidence interval with a *P* value of 0.047, 0.023 and 0.013 for compressive, flexural, and splitting tensile strength respectively which is less than the critical value of 0.05. This analysis offers valuable insights for optimizing mix designs and enhancing the overall performance and durability of laterized concrete in various construction applications^82^.

**Table 8:** ANOVA for compressive strength response.

Source	DF	Adj SS	Adj MS	F-value	*P*-value
Regression	4	211.937	52.9843	6.61	0.047
Slump	1	22.897	22.8972	2.85	0.166
w/c	1	65.506	65.5059	8.17	0.046
Laterite	1	0.000	0.0000	0.00	0.999
Granite size	1	0.612	0.6120	0.08	0.796
Error	4	32.083	8.0207		
Total	8	244.020

**Table 9:** ANOVA for flexural strength response.

Source	DF	Adj SS	Adj MS	F-value	*P*-value
Regression	4	2.80503	0.701257	10.07	0.023
Slump	1	0.31472	0.314717	4.52	0.101
w/c	1	0.80959	0.809590	11.63	0.027
Laterite	1	0.00828	0.008276	0.12	0.748
Granite size	1	0.00006	0.000055	0.00	0.979
Error	4	0.27853	0.069632		
Total	8	3.08356

**Table 10:** ANOVA for splitting tensile strength response.

Source	DF	Adj SS	Adj MS	F-value	*P*-value
Regression	4	1.46505	0.366263	13.93	0.013
Slump	1	0.14239	0.142385	5.42	0.080
w/c	1	0.46058	0.460580	17.52	0.014
Laterite	1	0.00090	0.000898	0.03	0.862
Granite size	1	0.00150	0.001505	0.06	0.823
Error	4	0.10515	0.026287		
Total	8	1.57020			

Table 11 provides a summary of the developed regression model, displaying coefficients of determination of 86.5%, 90.97%, and 93.30% for compressive, flexural, and splitting tensile strength respectively. These values signify strong prediction capabilities, indicating the percentage of variation in the response explained by the regression model outlined in Table 12.

**Table 11:** Regression model summary.

S	R-sq	R-sq(adj)	R-sq(pred)
Compressive strength			
2.83208	86.85%	73.70%	35.83%
Flexural strength			
0.263879	90.97%	81.93%	56.98%
Splitting tensile strength			
0.162133	93.30%	86.61%	69.87%

**Table 12:** Multiple linear regression equations.

Comp str.	=	53.6 + 0.196 (Slump) − 69.1 (w/c) − 0.001 (Laterite) + 0.176 (Granite size)
Flex. Str.	=	7.87 + 0.0230 (Slump) − 7.68 (w/c) + 0.0233 (Laterite) + 0.0017 (Granite size)
Split tensile	=	5.79 + 0.01548 (Slump) − 5.79 (w/c) + 0.0077 (Laterite) + 0.0087 (Granite size)

**Figure 13:**
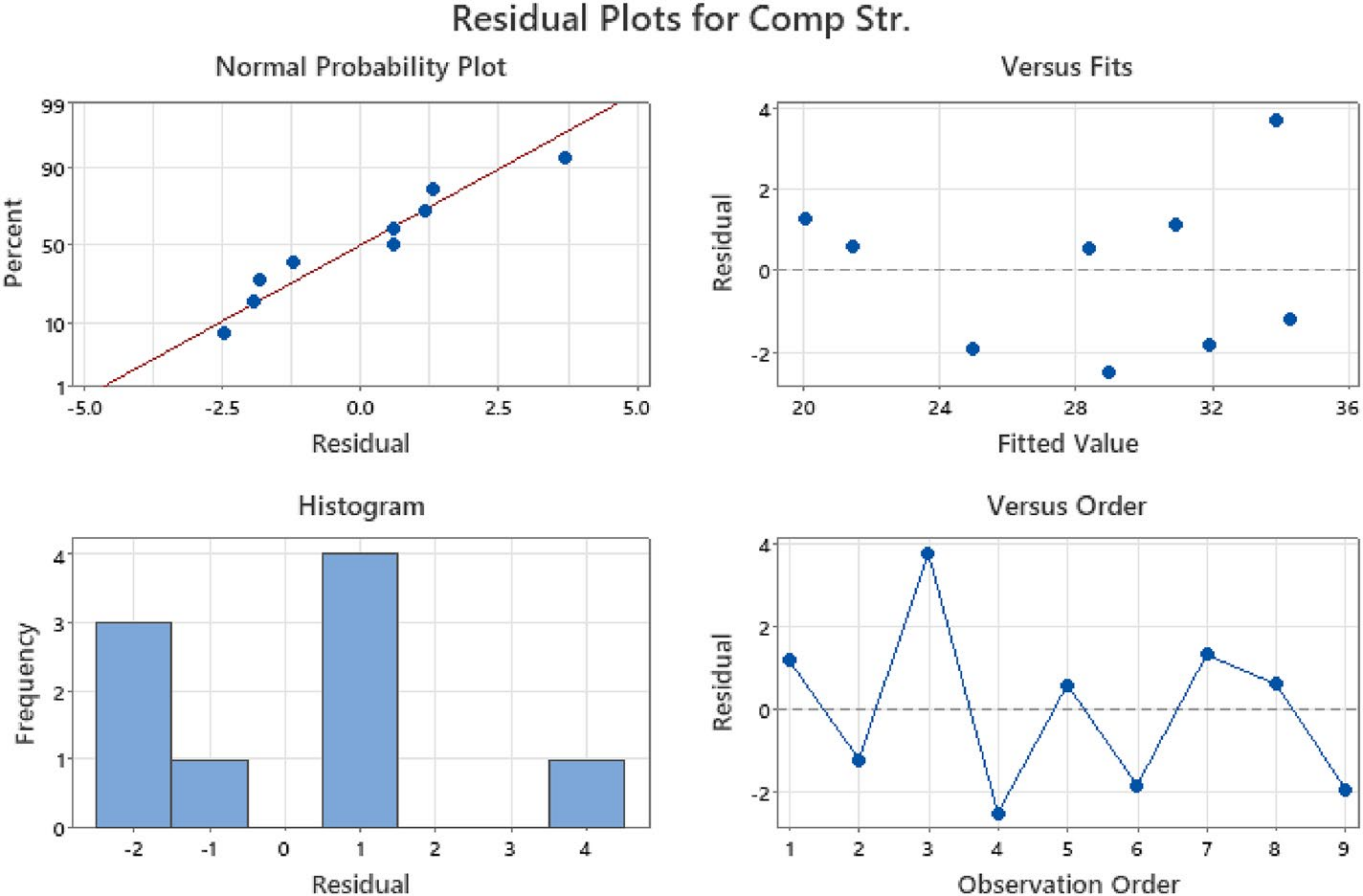
MLR residual plot for compressive strength.

**Figure 14:**
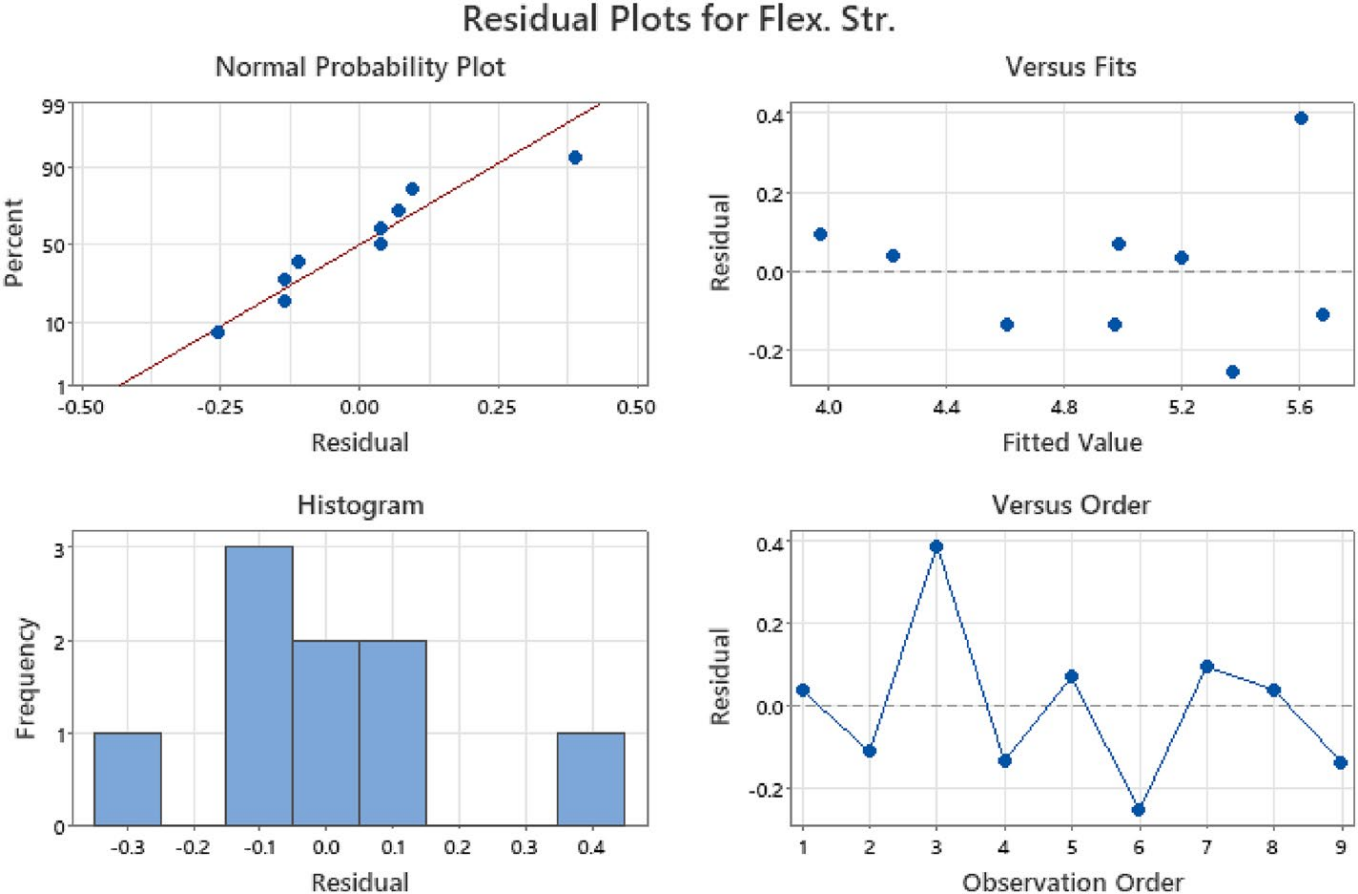
MLR residual plot for flexural strength.

**Figure 15:**
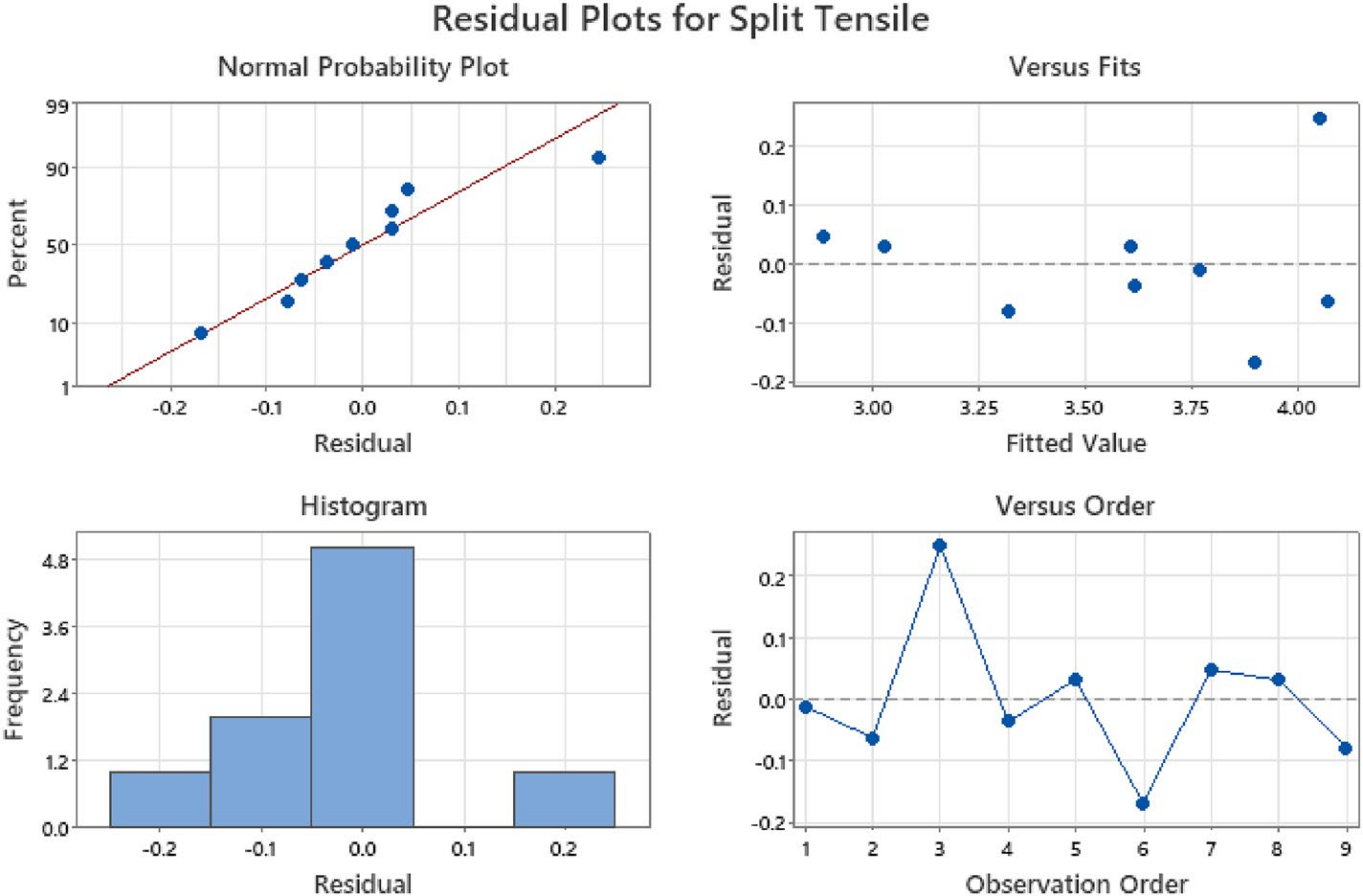
MLR residual plot for splitting tensile strength.

Figures 13, 14, and 15 display the residual plots for multiple linear regression (MLR) of compressive, flexural, and splitting tensile strength responses respectively. These graphs validate the fulfillment of regression model assumptions by depicting normal probability plots of residuals in percentage, fitted values against residuals within the range of − 1.0 to 1.0, frequency distribution histograms of residuals, and residuals plotted against observation order in percentage^83,84^. The findings from the statistical analysis indicated that water-cement ratio factor as the significant variable affecting the compressive, flexural, and splitting tensile strength responses, with p-values of 0.046, 0.027 and 0.014 respectively. The obtained results were in consonance with the findings of Ezeokpube et al*.*^56^ and Tam et al*.*^85^. To accurately predict the mechanical properties of concrete.

## Conclusion

The study on the effects of aggregate sizes on the structural properties of laterized concrete provides valuable insights into the behavior of concrete mixtures containing laterite as a partial replacement for fine aggregate. The following conclusions can be drawn from the experimental findings:

i. The workability of the concrete diminishes as the proportion of laterite in the concrete mix increases. To meet design standards, concrete should have slump values between 30 and 60 mm and a consistent water-cement ratio of 0.5. When maintaining this ratio, concrete samples without laterite showed better workability. This trend continued with 10% laterite content. However, to achieve a minimum 30 mm slump with 25% laterite replacing river sand, the water-cement ratio needed to be increased to 0.6. Additionally, larger coarse aggregates reduced workability.
ii. Larger coarse aggregates enhance the strength of laterized concrete, positively impacting compressive, flexural, and splitting tensile strength. Well-graded, 40 mm maximum coarse aggregates consistently yielded the highest strength across all tests, with 12 mm maximum size aggregates showing the lowest strength.
iii. The compressive strength of laterized concrete increased with increasing curing age. This suggests that longer curing periods result in improved strength properties because of pozzolanic reaction which occurs at prolonged hydration periods, a crucial consideration in construction projects where concrete strength development over time is important.
iv. The introduction of laterite as a partial substitute for fine aggregate influenced concrete's mechanical attributes. Higher laterite content led to decreased compressive, flexural, and splitting tensile strengths. Nonetheless, the concrete maintained satisfactory strength, surpassing the 25 N/mm^2^ characteristic strength requirement for 0% and 10% laterite replacement but falling short at 25%.
v. Adding laterite as a partial substitute for fine aggregate impacted concrete's mechanical properties. As laterite proportion increased, strength declined, yet remained acceptable for construction. While 0% and 10% laterite surpassed the 25 N/mm^2^ strength requirement, 25% fell short. Additionally, laterite addition slightly increased water absorption but maintained reasonable permeability for structural use.
vi. Regression analysis was employed to develop predictive models for the concrete's mechanical properties based on aggregate sizes, slump and laterite content. The models demonstrated a high coefficient of determination (R-squared), indicating their effectiveness in predicting concrete strength with result of 86.85%, 90.97%, 93.30% for compressive, flexural and splitting tensile strength responses respectively. Additionally, the developed predictive models can serve as valuable tools for optimizing concrete mix designs to achieve desired strength characteristics.

### Contribution to knowledge

By addressing the relationship between aggregate sizes and the performance of laterized concrete, the study adds valuable knowledge that can inform construction practices, enhance material selection, and promote the use of sustainable alternatives in the building industry. The research study makes valuable contributions to the existing knowledge in several ways:

i. The research provides insights into the influence of aggregate sizes on the mechanical properties of laterized concrete.
ii. It establishes that the size of aggregates plays a crucial role in determining the compressive strength, flexural strength, and splitting tensile strength properties of laterized concrete.
iii. The study employs statistical tools, such as Minitab and Microsoft excel, for regression analysis, showcasing a methodological contribution in assessing the impact of laterite on concrete strength.

### Recommendations for future study

The following recommendations for future research scope and restrictions are drawn from the results of this experimental investigation:

i. *Long-Term Durability Analysis***:** Assessment of the long-term durability and structural behavior of laterized concrete under environmental conditions and extended load cycles to ensure sustained performance.
ii. *Optimize Mix Proportions***:** Utilization of advanced modeling to optimize mix proportions, considering combinations of aggregate sizes for enhanced overall performance using artificial intelligence techniques.
iii. *Field Application Studies***:** Conduct field studies to assess real-world performance, challenges, and practical implications of laterized concrete with different aggregate sizes in construction projects.
iv. *Incorporate Other Additives***:** Study the interaction of aggregate sizes with additives like pozzolanic materials or chemical admixtures to enhance the properties of laterized concrete.
v. *Perform Economic Analysis***:** Conduct a cost–benefit analysis considering material costs, construction efficiency, and long-term maintenance to determine the economic feasibility of various aggregate sizes.

## Data Availability

All data generated or analyzed during this study are included in this published article.
